# Effect of intensive care after cardiac arrest on patient outcome: a database analysis

**DOI:** 10.1186/cc13847

**Published:** 2014-04-29

**Authors:** Andreas Schober, Michael Holzer, Helene Hochrieser, Martin Posch, Rene Schmutz, Philipp Metnitz

**Affiliations:** 1 Department of Emergency Medicine, Medical University of Vienna, Waehringer Guertel 18-20, A-1090, Vienna, Austria; 2 Section for Medical Statistics, Center for Medical Statistics, Informatics and Complex Systems, Medical University of Vienna, Spitalgasse 23, A-1090, Vienna, Austria; 3 Department of Anaesthesia and General Intensive Care Medicine, Medical University of Vienna, Waehringer Guertel 18-20, A-1090, Vienna, Austria

## Abstract

**Introduction:**

The study aimed to determine the impact of treatment frequency, hospital size, and capability on mortality of patients admitted after cardiac arrest for postresuscitation care to different intensive care units.

**Methods:**

Prospectively recorded data from 242,588 adults consecutively admitted to 87 Austrian intensive care units over a period of 13 years (1998 to 2010) were analyzed retrospectively. Multivariate analysis was used to assess the effect of the frequency of postresuscitation care on mortality, correcting for baseline parameters, severity of illness, hospital size, and capability to perform coronary angiography and intervention.

**Results:**

In total, 5,857 patients had had cardiac arrest and were admitted to an intensive care unit. Observed hospital mortality was 56% in the cardiac-arrest cohort (3,302 nonsurvivors). Patients treated in intensive care units with a high frequency of postresuscitation care generally had high severity of illness (median Simplified Acute Physiology Score (SAPS II), 65). Intensive care units with a higher frequency of care showed improved risk-adjusted mortality. The SAPS II adjusted, observed-to-expected mortality ratios (O/E-Ratios) in the three strata (<18; 18 to 26; >26 resuscitations per ICU per year) were 0.869 (95% confidence interval, 0.844 to 894), 0.876 (0.850 to 0.902), and 0.808 (0.784 to 0.833).

**Conclusions:**

In this database analysis, a high frequency of post-cardiac arrest care at an intensive care unit seemed to be associated with improved outcome of cardiac-arrest patients. We were able to identify patients who seemed to profit more from high frequency of care, namely, those with an intermediate severity of illness. Considering these findings, cardiac-arrest care centers might be a reasonable step to improve outcome in this specific population of cardiac-arrest patients.

## Introduction

Cardiac arrest occurs in 375,000 adults in Europe every year. Overall survival to hospital discharge ranges from 8% to 10% for out-of-hospital cardiac arrest, and is most commonly reported to be around 20% for in-hospital cardiac arrest [1-4]. Several factors (patient related and resuscitation related) have been identified to have considerable impact on outcome. Cardiopulmonary resuscitation of good quality, with uninterrupted chest compressions as well as early defibrillation (depending on initial electrocardiogram rhythm) significantly improves the outcome after cardiac arrest [5-7]. The etiology of cardiac arrest is also known to influence the prognosis. Of note is the fact that the outcome of patients resuscitated from cardiac arrest treated in different hospitals and intensive care units (ICUs) has shown a significant variability [8-10].

Hospital factors during the postresuscitation period and their implications for patient survival have long been underappreciated and have not been well defined. Geographic factors as well as the level of care of the centers involved influence outcome after cardiac arrest and cost effectiveness [9,11,12]. The first standardized algorithms for the postresuscitation-care period have just recently been implemented in the European guidelines for resuscitation [13]. Several hospital-related factors, such as hospital size and teaching status, have been identified to be of importance for the outcome after cardiac arrest, and regional differences have been described [10,14,15]. With regard to the role models of acute coronary care units, stroke-, trauma-, and burn-injury centers, specialized cardiac arrest-care centers, as well as predefined treatment bundles for the postresuscitation period have become a subject of discussion recently [16-24].

The aim of this study was to investigate the impact of ICU-related factors and ICU characteristics on the patient’s outcome after cardiac arrest. We furthermore suspected that the frequency of patients treated after cardiac arrest, the hospital size, and the capability to perform coronary angiography and intervention influence cardiac arrest mortality.

## Methods

The Austrian Centre for Documentation and Quality Assurance in Intensive Care Medicine (ASDI), a nonprofit organization that has established an intensive care database and benchmarking project in Austria, prospectively collected intensive care unit (ICU) data. The collected data included demographic background information, such as age, sex, and preexisting chronic conditions (comorbidities); the reasons for ICU admission that were recorded according to a list of medical and surgical diagnoses [25]; severity of illness according to the Simplified Acute Physiologic Score (SAPS II), determined at admission; level of provided care, as measured by the Simplified Therapeutic Intervention Scoring System (TISS-28) [26]; length of ICU and hospital stay; and status at ICU and hospital discharge (survival/death).

The study protocol, and waiving of informed consent (no interventions were performed, and no individual data were analyzed) were approved by the Ethics Committee of the Medical University of Vienna.

To assess the reliability of data collection, interobserver variability was checked at regular intervals. Variance-component analyses with the random factors “units,” “patients within units,” and “observers within units” were performed as described previously [27]. To assess the completeness of the documentation, the number of missing parameters for the SAPS II score was calculated. Additional details have been reported elsewhere [27].

All patients who were continuously admitted to 87 Austrian ICUs between 1998 and 2010 were evaluated for this study (*n* = 279937). From these, patients without a unique identifier as well as patients who were documented twice were excluded (*n* = 366). For patients who were admitted more than once (*n* = 19,426), only the first admission was included. Patients who were younger than 18 years (*n* = 5,386), those with records that lacked an entry in the field “hospital outcome” (*n* = 2,108), and those without a valid SAPS II score (*n* = 10,063) were also removed. Of this sample (*n* = 242,588), a cohort of 5,857 patients fulfilled the inclusion criterion of resuscitation as the main diagnosis at admission to the ICU.

Statistical analysis was performed by using the SAS software version 9.2 (SAS Institute Inc., Cary, NC, USA). For tests of statistical significance, ANOVA for normally distributed data and the Kruskal-Wallis test for distorted data were used. Furthermore, the χ^2^ test was used when appropriate. A *P* value of < 0.05 was considered significant. Unless otherwise specified, descriptive results are expressed as median and first and third quartiles, respectively. Risk-adjusted mortality was calculated by dividing the number of observed deaths per group by the number of SAPS II-predicted deaths per group. To identify risk factors for hospital mortality, univariate logistic regressions were performed.

The primary analysis to investigate the association of frequency of intensive care after cardiac arrest and mortality was a multivariate generalized estimating equations analysis (SAS Proc Genmod) accounting for correlations within ICUs with dependent variable, hospital mortality, and independent factors of postresuscitation care frequency, SAPS II score, an interaction term of resuscitation-care frequency and SAPS II, gender, number of ICU beds, calendar year (calendar years were dichotomized into two groups, before and after the year 2005), ICU type (medical, postoperative surgical intensive care unit in hospital ≤500 beds, postoperative surgical intensive care unit in hospital >500 beds, Trauma). Variables that are part of the SAPS II score (for example, age, GCS) were not included separately in this analysis.

To illustrate the influence of the annual case load on hospital mortality, mortality rates were plotted stratified by the number of resuscitations per ICU per year (<18; 18 to 25; ≥26 resuscitations per ICU per year) and SAPS II score (divided into intervals with width 20). Furthermore, the distribution of risk factors in Low, Medium, and High treatment-frequency ICUs, as defined earlier, was compared.

## Results

Hospital mortality after cardiac arrest and risk factors are displayed in Table 1. Chronic renal insufficiency, chronic heart failure, chronic respiratory failure, diabetes mellitus, liver cirrhosis, malignant disease, and hematologic disease are associated with hospital mortality. For all of these comorbidities, the mortality is higher (odds ratios between 1.3 and 2.8). Differences in baseline factors of the patients treated in the three different strata of ICUs are shown in Table 2.

**Table 1:** Univariate/multivariate testing: mortality-associated factors of patients admitted after cardiac arrest

	**Univariate logistic regression**			**Multivariate generalized estimation equation model**	
	**All patients (died in hospital, %)**	**Odds ratio (95% CI)**	***P *** **value**	**Odds ratio (95% CI)**	***P *** **value**
SAPS II score^a^		1.061 (1.057-1.064)	<0.001	1.05 (1.05-1.06)	<0.001
Male	3,753 (54.92)	0.85 (0.76-0.95)	0.003	0.96 (0.84-1.09)	0.5262
Age		1.03 (1.02-1.03)	<0.001		
Year		1.01 (0.99-1.02)	0.241	0.98 (0.96-1.01)	0.187
**Year dichotomized (before and after 2005)**		1.065 (0.96-1.181)	0.237	0.87 (0.73-1.04)	0.1386
**Comorbidities**					
Chronic renal insufficiency	618 (66.83)	1.64 (1.37-1.95)	<0.001		
Chronic respiratory insufficiency	510 (63.92)	1.41 (1.17-1.7)	<0.001		
Chronic cardiac failure NYHA^b^ IV	1,474 (61.67)	1.34 (1.19-1.51)	<0.001		
Coronary angiography unit in hospital	3,805 (56.64)	0.93 (0.82-1.04)	0.205		
**ICU type**					
Medical cardiac	1,739 (58.48)	Reference		Reference	
Medical	1,850 (55.84)	0.9 (0.79-1.02)	0.110	0.79 (0.59-1.07)	0.1256
Postoperative:– hospital <500 beds	1,172 (59.3)	1.03 (0.89-1.2)	0.660	0.82 (0.61-1.1)	0.1858
Postoperative: hospital ≥500 beds	1,008 (51.98)	0.77 (0.66-0.9)	0.001	0.76 (0.5-1.13)	0.1767
Trauma	88 (37.5)	0.43 (0.27-0.66)	<0.001	0.39 (0.17-0.92)	0.0313
**Admission type**					
Medical	5,665 (56.88)	Reference			
Scheduled surgery	88 (28.41)	0.3 (0.19-0.48)	<0.001		
Unscheduled surgery	95 (49.47)	0.74 (0.49-1.11)	0.147		
10 resuscitations per year		0.99 (0.95-1.03)	0.497	0.7 (0.57-0.86)	0.0009
SAPS II × 10 resuscitations per year				1 (1–1.01)	0.014
Beds in ICU		0.96 (0.95-0.98)	<0.001	1.02 (0.96-1.08)	0.4823
**TISS-intervention (patient received: yes/no)**					
Mechanical ventilation	5,339 (60.09)	6.79 (5.39-8.55)	<0.001		
Enteral nutrition	2,962 (49.66)	0.57 (0.52-0.64)	<0.001		
Parenteral nutrition	2951 (53)	0.76 (0.68-0.84)	<0.001		
Renal support	482 (68.88)	1.79 (1.47-2.19)	<0.001		
Single vasoactive medication	3,489 (52.08)	0.65 (0.58-0.72)	<0.001		
Multiple vasoactive medication	2,803 (64.4)	1.88 (1.69-2.09)	<0.001		
Interventions outside the ICU	2,738 (50.26)	0.63 (0.56-0.69)	<0.001		
Cardiopulmonary resuscitation	2,704 (68.9)	2.64 (2.37-2.94)	<0.001		

Odds ratios were constructed for hospital mortality with univariate/multivariate analysis. ^a^SAPS II, Simplified Acute Physiology Score II; ^b^NYHA, New York Heart Association functional classification.

**Table 2:** Cardiac arrest treatment frequency strata and patients baseline characteristics

	**Low-treatment-frequency ICU**^**a**^	**Medium-treatment-frequency ICU**^**a**^	**High-treatment frequency ICU**^**a**^	***P *** **value**
Number of patients	1,986 (33.9)	2,013 (34.4)	1,858 (31.7)	
Age in years	72.0 (61.0 - 80.0)	70.0 (60.0 - 78.0)	69.0 (58.0 - 78.0)	<0.001
Sex female	806 (40.7)	685 (34.1)	607 (32.7)	<0.001
SAPS II^b^ score	63 (49;78)	62 (49;75)	65 (54;78)	<0.001
Medical admission	1,845 (92.0)	1,995 (99.5)	1,825 (98.3)	<0.001
Scheduled surgical	70 (3.5)	6 (0.3)	12 (0.7)	<0.001
Unscheduled surgical	71 (3.6)	5 (0.3)	19 (1.0)	<0.001
Chronic renal insufficiency	233 (11.7)	190 (9.4)	195 (10.5)	0.0614
Chronic respiratory insufficiency	225 (11.3)	117 (5.8)	168 (9.0)	<0.001
Chronic cardiac failure NYHA^c^ IV	517 (26.0)	436 (21.7)	521 (28.0)	<0.001
Liver cirrhosis	68 (3.4)	49 (2.4)	53 (2.9)	0.1738
Insulin-dependent diabetes mellitus	147 (7.4)	134 (6.7)	156 (8.4)	0.1195

Displayed as median with IQR (interquartile range from 25^th^ to 75^th^ quartile), frequencies are displayed in absolute numbers (*n*) and (%). ^a^ICU, Intensive Care Unit; ^b^SAPS II, Simplified Acute Physiology Score II; ^c^NYHA, New York Heart Association functional classification.

Observed hospital mortality was 56% in the cardiac-arrest cohort. Observed ICU mortality was 45% with 2,649 nonsurvivors. SAPS II predicted mortality was 66%. Risk-adjusted mortality was 0.851 (0.836 to 0.865) for cardiac-arrest patients.

Table 3 shows the measures of intensive care as reflected by the TISS-28 score for each of the three strata of ICUs grouped by cardiac-arrest frequency. In high-frequency cardiac arrest ICUs, enteral nutrition was favored over parenteral nutrition. Renal support was initiated in fewer patients in ICUs with high case loads of postresuscitation patients. Patients received fewer diuretics in high-frequency ICUs. A combination of vasopressor medications was applied in fewer patients in ICUs with a high frequency of postresuscitation care. A peripheral artery catheter was used in more patients in high-frequency ICUs.

**Table 3:** **Interventions as measured by TISS-28**^**a **^**score parameters by frequency of postresuscitation care in the ICU**

	**Low-treatment-frequency ICU**^**b**^	**Medium-treatment-frequency ICU**^**b**^	**High-treatment-frequency ICU**^**b**^	***P *** **value**
TISS-28^a^ score per patient per day; median (IQR^c^)	33.5 (28.7; 38.3)	32 (26; 36.2)	33.3 (29.7;3 7.2)	<0.001
	*n* (% of patients)	*n* (% of patients)	*n* (% of patients	
Ventilatory support	802 (40.38)	995 (49.43)	823 (44.29)	<0.001
Mechanical ventilation	1,837 (92.5)	1,714 (85.15)	1,788 (96.23)	<0.001
Enteral nutrition	981 (49.4)	881 (43.77)	1,100 (59.2)	<0.001
Parenteral nutrition	1,024 (51.56)	945 (46.94)	982 (52.85)	<0.001
Renal support	170 (8.56)	189 (9.39)	123 (6.62)	0.006
Routine dressing changes	1,520 (76.54)	1,813 (90.06)	1,585 (85.31)	<0.001
Frequent dressing changes	424 (21.35)	439 (21.81)	230 (12.38)	<0.001
Diuretics treatment	1,031 (51.91)	922 (45.8)	722 (38.86)	<0.001
Single vasoactive medication	1,197 (60.27)	1,216 (60.41)	1,076 (57.91)	0.2108
Multiple vasoactive medications	1,021 (51.41)	892 (44.31)	890 (47.9)	<0.001
Multiple intravenous medications	1,865 (93.91)	1,952 (96.97)	1,779 (95.75)	<0.001
Peripheral arterial catheter	1,719 (86.56)	1,534 (76.2)	1,674 (90.1)	<0.001
Care of drains	834 (41.99)	406 (20.17)	414 (22.28)	<0.001

Frequencies are displayed in absolute numbers (*n*) and (%). ^a^TISS-28, Therapeutic Intervention Scoring System; ^b^ICU, intensive care unit; ^c^IQR, interquartile range of 25^th^ to 75^th^ quartile.

Comparing outcome in different ICUs, we found a lower mortality for ICUs in a hospital with more than 500 beds, but it was not significant in the multivariate analysis. The existence of a coronary angiography unit at the hospital was not significantly associated with outcome. Results are displayed in Table 1.

The unadjusted univariate analysis did not show a significant association of hospital mortality and the frequency of resuscitation care at the ICU (OR, 0.99; CI, 0.95 to 1.03, *P* value = 0.497), but the frequency of post-cardiac arrest intensive care was associated with risk-adjusted mortality. The ICU stratum with the highest number of resuscitations showed the lowest risk-adjusted mortality. The SAPS II adjusted, observed-to-expected mortality ratios (O/E-Ratios) in the three strata (<18; 18 to 26; more than 26 resuscitations per ICU per year) were 0.869 (95% CI, 0.844 to 894), 0.876 (0.850 to 0.902), and 0.808 (0.784 to 0.833).

**Figure 1:**
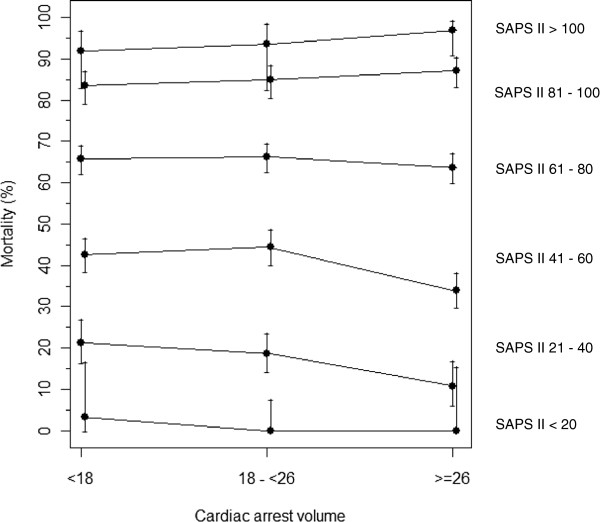
**Association between mortality, severity of illness, and frequency of post-cardiac arrest intensive care provided in an ICU.** x-axis: Frequency of patients treated after cardiac arrest, divided into tertiles. y-axis, Mortality ± 95% confidence intervals; SAPS II is divided into steps by 20: <20 (lowermost line), <40, <60, <80, <100, ≥100 (topmost line). Every second line (+CI) is shifted for improved identification of the confidence intervals.

The results of multivariate analysis are displayed in Table 1. SAPS II score was significantly associated with hospital mortality and the frequency of postresuscitation care at the ICU.The interaction of the frequency of postcardiac arrest intensive care with SAPS II score showed that, for patients with high SAPS II scores, the dependency of mortality on the frequency of resuscitations is lower (the corresponding interaction term “SAPS II × 10 patients per year” showing an odds ratio >1). This interesting result is displayed in Figure 1, which shows the association between crude hospital mortality, severity of illness, and the frequency of postresuscitation care in a diagram. For patients with high SAPS II scores, mortality is constant, despite increasing frequency of cardiac-arrest care, whereas, for patients with lower to medium SAPS II scores, it shows a falling tendency with increasing case load.

## Discussion

We evaluated the association between patient-related factors, comorbidities, intensive care measures, and their impact on the outcome for patients treated after cardiac arrest in different ICUs. Significant outcome differences occurred between different ICUs that were treating cardiac-arrest patients. In high-frequency ICUs, we found a similar mortality, although patients in these ICUs had significantly higher predicted mortality.

The need for treatment bundles for postresuscitation care and the foundation of cardiac-arrest centers have recently been discussed intensively [18]. Several studies have shown that outcome after cardiac arrest is influenced by postresuscitation care measures and the treating facility itself.

A comparison of postresuscitation care in Göteborg found a significant difference in survival-to-discharge rate of two hospitals (33% versus 44%). This was caused by baseline differences, socioeconomic status, and in-hospital factors (technical capabilities and staff resources) [9].

Carr *et al.*[14] investigated correlations between hospital-related factors and outcome after cardiac arrest in different hospitals in the United States. They found different mortality rates by comparing hospital status than by using treatment frequency. The highest survival was found in large teaching hospitals in urban areas [14]. The same authors found an association between the volume of cardiac-arrest cases treated per year and favorable outcome [10].

Langhelle *et al.*[11] compared in-hospital factors influencing outcome after out-of-hospital cardiac arrest in four different regions in Norway. They found significant differences in survival to discharge, linked to Utstein-related out-of-hospital and in-hospital-factors. However, they did not compare hospital capacities and cardiac-arrest treatment frequencies.

In contrast, Callaway *et al.*[12] found no independent associations between survival or length of stay and hospital characteristics.Our results, though, add further evidence to support the initiation of cardiac-arrest care centers. Even though crude mortality of cardiac-arrest patients did not differ between the ICUs stratified by treatment frequency, we found a significant decrease in risk-adjusted mortality when correcting for factors such as the severity of illness, the year, ICU specialty, patient sex, age, and comorbidities, hospital size, as well as the number of ICU beds. Our analysis identified a subgroup of cardiac-arrest patients with low to intermediate SAPS II scores, whose postcardiac-arrest intensive care in a specialized high-frequency center was associated with lower mortality (Figure 1). This might be caused by the fact that most patients above a specific SAPS II score are so severely injured that none of the expertise in those high-frequency centers could change their grim outcome.

We also tried to identify the intensive care-specific factors that differed between ICUs with high and low frequency of postcardiac-arrest care. A significant difference in intensive care measures provided was found. The reduced use of mechanical ventilation, renal support, and multiple vasoactive substances probably reflects a different treatment approach in higher-volume centers. In contrast to Callaway *et al.*[12], we did not find an association between the hospital’s capability to perform coronary angiography and mortality.

Because severity of illness was significantly higher in patients treated in high-volume centers, we expected those measures to have been provided more frequently.

This study is limited by the fact we were not able to provide data of the resuscitation process and the cause of cardiac arrest according to Utstein criteria [28], because the ASDI database was not conducted as a cardiac-arrest registry. Conversely, the size of the database allowed detailed comparisons of in-hospital treatment factors in this cohort.

Another limitation with respect to outcome evaluation is the lack of data concerning the use of therapeutic hypothermia and the performance of coronary intervention. Furthermore, assessment of neurologic performance at the time of hospital discharge as well as neurologic follow-up according to Utstein criteria, was not performed. Additionally, the analysis was retrospective and performed only in ICUs willing to provide their data to the database, thus introducing the possibility of a significant selection bias.

Some of the factors that influence treatment of patient after cardiac arrest might have been missed. Conversely, the TISS-28 scores provided a good measure of actual efforts. Withdrawal of care was not recorded in the database. Therefore, it might be possible that ICU mortality was influenced by differences in withdrawal-of-care rates. As withdrawal of care is, in most of the cases, a patient-specific decision, we think that the size of the database was able to balance out possible differences.

## Conclusions

In this database analysis, a high frequency of postcardiac-arrest care at an intensive care unit seemed to be associated with improved outcome of cardiac-arrest patients. We were able to identify patients who seemed to profit more from high frequency of care: those with an intermediate severity of illness. Considering these findings, cardiac-arrest care centers might be a reasonable step to improve outcome in this specific population of cardiac-arrest patients.

## Key messages

• A high frequency of postcardiac-arrest care at an intensive care unit can improve the outcome of cardiac-arrest patients.

• Cardiac-arrest care centers must be implemented.

## Abbreviations

ANOVA: Analysis of variance; ASDI: Austrian Centre for Documentation and Quality Assurance in Intensive Care Medicine; CI: confidence interval; GCS: Glascow coma scale; ICU: intensive care unit; IQR: interquartile range; NYHA: New York Heart Association functional classification; O/E: Observed-to-expected ratio; OR: odds ratio; SAPS II: Simplified Acute Physiologic Score; TISS: Therapeutic Intervention Scoring System.

## Competing interests

The authors declare that they have no competing interests.

## Authors’ contributions

The study was planned and designed by AS, HH, MH, RS, and PM. PM and HH carried out the data acquisition. HH performed statistical analysis with critical revision and substantial contributions provided by AS, MH, PM, and MP. Interpretation of data was carried out mainly by AS, MH, MP, and PM. AS and MH drafted the manuscript, with substantial contributions of HH, RS, MP, and PM. All authors read, provided critical revision to, and approved the final manuscript. All authors agree to be accountable for all aspects of the work in ensuring that questions related to the accuracy or integrity of any part of the work are appropriately investigated and resolved. Each author has participated sufficiently in the work to take public responsibility for appropriate portions of the content.
